# Deep Reads: Strands in the History of Molecular Genetics

**DOI:** 10.1371/journal.pgen.1004887

**Published:** 2014-12-18

**Authors:** Andrew D. Chisholm

**Affiliations:** Division of Biological Sciences, University of California San Diego, La Jolla, California, United States of America

*Mr. Elphinston talked of a new book that was much admired, and asked Dr. Johnson if he had read it. JOHNSON. 'I have looked into it.' 'What, (said Elphinston,) have you not read it through?' Johnson, offended at being thus pressed, and so obliged to own his cursory mode of reading, answered tartly, 'No, Sir, do* you *read books* through?'—James Boswell, The Life of Samuel Johnson

 

*“Reading rots the mind”—sign in Francis Crick's office*

The history of molecular genetics, its precursors and descendants, is rich in events and colorful characters intertwining through the tumultuous history of the mid-20th century. In this second iteration of “Deep Reads,” I try to trace selected strands in the ancestry of the field as told in histories, biographies, and memoirs, some of which I have, unlike Johnson, read through (Image 1).

**Figure:**
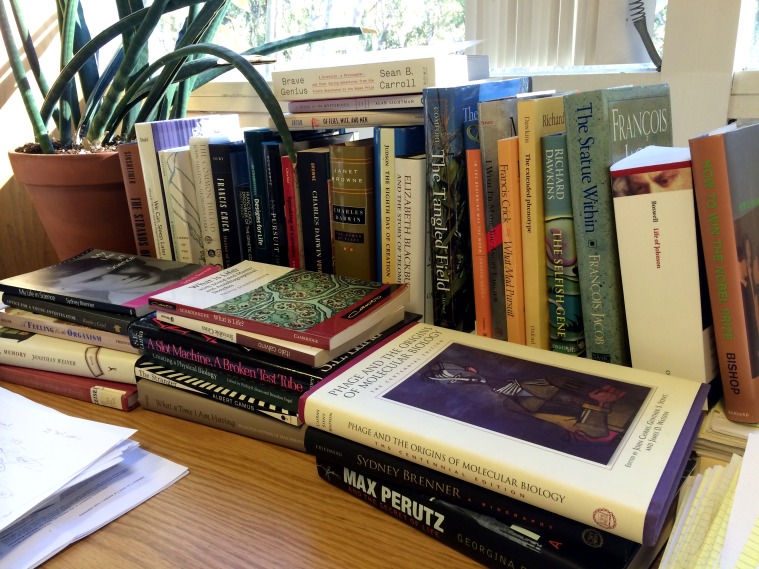
Some books consulted in the writing of this piece. Photo courtesy of Andrew D. Chisholm.

These recommendations form a tale of three successive intellectual utopias. In the first act, the precursors of molecular genetics take hold among physicists, in particular in those around Niels Bohr. In the second act, molecular genetics emerges in the 1940s, spreading out from the phage group and together with structural biology forming the nascent field of molecular biology. In the third act, a diaspora generation of geneticists applies the style of phage genetics to a menagerie of organisms: *Caenorhabditis elegans*, *Drosophila*, zebrafish, and *Arabidopsis*, among others.

First though, for an epic and accessible history of molecular biology and genetics, I, like Jane Gitschier in the 2013 “Deep Reads,:Recommendations from Jane Gitschier′s Bookshelf ” return again to Horace Freeland Judson's ***The Eighth Day of Creation*** (1979, expanded edition in 1996). I first read this as a teenager and remain captivated by Judson's crisp, drama-filled accounts of discovery, based on countless interviews with the participants. As well as recounting the eureka moments, Judson also gives us the twists and turns, the blind alleys, and the failed experiments. A high point is the story of the maelstrom of experiment and theory that led to the deciphering of the genetic code (Part II, “RNA: The Functions of the Structure”). If you read one book on the history of modern genetics, this has to be it!

## Act I: Before *What Is Life?*

Many of those interviewed by Judson traced their interest in biology to reading Erwin Schrödinger's ***What Is Life?*** (1943), which was based on lectures given at Trinity College Dublin. This influential treatise, still highly readable, gives the first encapsulation of the duality of the gene as a physical structure (the “aperiodic solid”) and as a “miniature code.” Not everyone has had the same high opinion: Max Perutz commented that “what was true in his [Schrödinger's] book was not original, and most of what was original was known not to be true.” Nevertheless, *What Is Life?* inspired many to start thinking about the physical nature of genes, in large part by bringing Delbrück's model of the gene to the attention of a larger audience.

What was Delbrück's model? To find out we have to go back to the fabled *Dreimännerwerk*, or “Three-Man Paper” (3MP), of Nikolai Timoféev-Ressovsky, Karl Zimmer, and Max Delbrück, published in the Proceedings of the Göttingen Academy of Sciences in 1935 and for many years well-nigh impossible to find, in English at least. An excellent scholarly translation is now available in ***Creating a Physical Biology: The Three-Man Paper and Early Molecular Biology*** (2011, eds. Phillip R. Sloan and Brandon Fogel), along with essays placing the paper in its context as the urtext of molecular biology. At the time, the rise of fascism already loomed: the 3MP arose from informal seminars at the Delbrück family home in Berlin, held for those recently expelled from their research posts by the Nazis. The 3MP rests on a quantitative analysis of radiation mutagenesis in *Drosophila*; while superseded in many places, the 3MP is well worth rereading for its analytic rigor and its formulation of a gene as a “physical-chemical unit.” The 3MP takes a remarkably sophisticated view of the roles of genes in development, rejecting simplistic ideas of genes as the ultimate units of life, in contrast to Schrödinger's more influential hardcore reductionism.

Delbrück was a theoretical physicist whose interest in biology was stimulated by Bohr's speculations on complementarity. For its wonderfully evocative account of the atmosphere of the Bohr circle and the quantum mechanics group, I enjoyed ***Faust in Copenhagen: A Struggle for the Soul of Physics*** (2007), by the physicist Gino Segrè. Bohr's Institute for Theoretical Physics in Copenhagen held famous annual meetings, of which perhaps the most legendary was that held in 1932, the “miracle year” of quantum mechanics and the centenary of Goethe's death. The book is structured around the entertainment at the meeting, a parody of Goethe's *Faust* devised by none other than the young Max Delbrück.

## Act II: War and Phage

Our middle act revolves around bacteriophage (and their bacterial hosts). Unknown until about 1915–1917, phage were not immediately seized on by physicists looking at biology: they are not mentioned in the 3MP or in *What Is Life?*. Delbrück first realized the importance of phage in 1936 in a chance conversation on a visit to the California Institute of Technology (CalTech) (to work with Morgan on *Drosophila*). But where did the phage come from? One starting point is with the life of Felix d'Herelle, codiscoverer of phage and a nomadic eccentric whose life story sounds stranger than fiction. d'Herelle was a self-taught microbiologist whose first published paper claimed that carbon was not an element. Early in his career, he was involved in ventures to distill whisky from maple syrup (in Canada), from rotting bananas (in Guatemala), and from the nonfibrous residue of sisal (in Mexico). In 1917 he discovered phage, became embroiled in priority claims with Frederick Twort, and then traveled the world using phage therapy as a cure for bacterial diseases. d'Herelle himself was a Lamarckian with no affinity for the reductionist geneticists who later took over his beloved phage. The biography ***Félix d'Herelle and the Origins of Molecular Biology*** (1999) by William Summers is a nice account of d'Herelle's wildly diverse pursuits and peregrinations.

d'Herelle, or at least his work, may have been in part the inspiration for ***Arrowsmith*** (1925) by Sinclair Lewis, the one work of fiction I will recommend here (and Seymour Benzer's http://en.wikipedia.org/wiki/Seymour_Benzer favorite book). The portrayal of Martin Arrowsmith, an obsessive idealist who gives up a lucrative medical career to follow his research dreams, may perhaps not be completely out of date. Arrowsmith was supposedly modeled after Paul de Kruif, author of the classic (but even more dated) ***Microbe Hunters*** (1926).

The career of Delbrück, his founding of the phage group (or “phage church”) and the development of molecular genetics as a discipline, has been told in several places, although Delbrück himself left no memoir. For a vivid depiction of the “voices of those involved,” the Festschrift volume ***Phage and the Origin of Molecular Biology*** (1966) remains essential reading. The essays by Seymour Benzer, Andre Lwoff, and Bob Edgar are lively mixtures of amusing recollections with serious science.

As a counterpoint to such Delbrückiana, it is worth seeking out Salvador Luria's autobiography, ***A Slot Machine, a Broken Test Tube*** (1984, now sadly out of print). Luria gives an engagingly down to earth and at times confessional account of his early life and his path to the momentous collaboration with Delbrück on the fluctuation test. (This paper contains one of the first and best author contributions: “Theory by M.D., experiments by S.E.L.”) Luria became the founding director of the Massachusetts Institute of Technology (MIT) Center for Cancer Research. What is striking now is how active Luria was in progressive politics, as a vocal opponent of the Vietnam War and a supporter of the labor movement. One wonders what Luria would make of the current MIT cancer center with its Koch sponsorship.

Alongside the phage church, bacterial genetics was entering its heroic postwar period, which included the emergence of the team of Andre Lwoff, François Jacob, and Jacques Monod at the Pasteur Institute in Paris. All had tumultuous experiences in war, Lwoff and Monod in the resistance and Jacob as a medical officer in the Free French Forces. Jacob's autobiography ***The Statue Within*** (*La statue intérieure*, 1987), written in a deliberately fragmented montage style, focuses on his early life: boyhood, medical training, wartime service in Africa, a near-fatal shrapnel wound, and his postwar quest to become a biologist.

Jacques Monod's wartime exploits and his later rise to scientific eminence have been grippingly recounted in Sean B. Carroll's double biography of Monod and Albert Camus, ***Brave Genius*** (2014). Monod and Camus both worked for the Resistance in Paris. Camus was the editor of the underground newspaper *Combat*; Monod (nom de guerre, Malivert) lived a double life, analyzing *Escherichia coli* growth curves by day and planning sabotage operations by night. Yet Camus and Monod did not meet until three years after the war, when Monod became well-known for his denunciation of the pseudoscience of Lysenkoism. As a molecular biologist and a World War II buff, Carroll expertly covers a vast range of events, people, and ideas.

In Cambridge in the late 1950s and early 1960s, phage geneticists, led by Sydney Brenner with a brief intervention by Francis Crick, used formal properties of mutagenesis in phage to decipher the genetic code. After reading Judson it is instructive to read Crick's sharply opinionated semimemoir, ***What Mad Pursuit: A Personal View of Scientific Discovery*** (1990). Crick intersperses science with brief autobiographical vignettes. During WWII, Crick worked on the design of naval magnetic mines, a period he passes over in his memoir but which is discussed in absorbing detail in the official biography, ***Francis Crick: Hunter of Life's Secrets***, by Robert Olby (2009). Crick's naval warfare experience, while far from biology, taught him many important lessons valuable for his later scientific career: the need for constant improvisation and critical re-evaluation, how to bypass bureaucracy, and the importance of creative rule-breaking. For a shorter introduction to Crick's life and work, Matt Ridley's breezy minibiography, ***Francis Crick: Discoverer of the Genetic Code*** (2006), despite a somewhat inaccurate title, is a good thumbnail sketch.

Crick and Brenner worked, and for many years shared an office, at the Medical Research Council's Laboratory of Molecular Biology (LMB) in Cambridge, whose chairman, Max Perutz, played an essential role in fostering the environment and institutional support of phage and later *C. elegans* genetics. Perutz was a pioneer of protein crystallography yet also a cultured and prolific writer whose essays and book reviews combine erudition, sensitivity, and a dry humor; some are collected in ***I Wish I'd Made You Angry Earlier*** (2002), complete with Perutz's “commonplace book” of inspiring quotations. As a Jewish refugee from Vienna, Perutz's wartime experience began with internment in a camp for enemy aliens in Canada. He was eventually released, going on to perform military research, most notably in the quixotic attempt to convert icebergs into floating airfields (Project Habbakuk). Max's witty letters are on a par with his essays and are collected in ***What a Time I am Having*** (2008). (For the Perutz completists, there is an excellent biography by Georgina Ferry, ***Max Perutz and the Secret of Life***, 2007).

For many molecular geneticists, WWII was *the* formative experience. In her fascinating study of Cambridge molecular biology, ***Designs for Life*** (2002), the historian of science Soraya de Chadarevian traces how the expansion of molecular biology (and thus molecular genetics) was an integral part of postwar reconstruction in Britain. A weighty academic tome, *Designs for Life* is scattered with intriguing footnotes, such as when Crick's application for the chair in genetics at Cambridge was turned down, on the grounds that he did not know any genetics. de Chadarevian emphasizes that the history of science is not simply the stories of a small circle of “hero figures,” either self-told or as told to journalists or official biographers, and she broadens the scope to the roles of technical staff, administrators, and the political milieu.

## Act III: The Diaspora

Delbrück described phage as “a fine playground for serious children who ask ambitious questions.” In the mid-1960s, many of the “serious children” began to ask their questions about development, the nervous system, and behavior. Some returned to classical organisms, such as *Drosophila*, adapting them for the more systematic genetic screens that were the secret to success. An early migrant from phage to *Drosophila* was Seymour Benzer, who almost single-handedly founded modern *Drosophila* neurogenetics. In ***Time, Love, Memory: A Great Biologist and His Quest for the Origins of Behavior*** (2000), the popular science writer Jonathan Wiener adeptly uses the biography of Benzer and his lab to explain the genetic basis of complex behaviors.

Other phage geneticists boldly explored new animal genetic models, including the nematode *C. elegans* (Sydney Brenner) and the zebrafish (George Streisinger). Not all of these ventures were successful; Delbrück himself spent the last quarter century of his career studying *Phycomyces*, to little avail. One success story (and admittedly, I am a bit biased here) was of course *C. elegans*. ***In The Beginning Was The Worm: Finding the Secrets of Life in a Tiny Hermaphrodite*** (Andrew Brown, 2003) is an appealingly chatty account of the development of the *C. elegans* field. Brown describes the pioneer days as “a remarkable story of altruism, cooperation, and general niceness,” and while there may be some mythologizing, some vestiges of the original communal spirit remain.

A central figure in the elucidation of *C. elegans* development and in the mapping and sequencing of its genome is John Sulston, who went on to lead the publicly funded Human Genome Project. We take it for granted that such genomic data is open access, yet during the public-private race to sequence the human genome in the 1990s, such an outcome was by no means assured. Sulston and Georgina Ferry's ***The Common Thread: A Story of Science, Politics, Ethics and the Human Genome*** (2002) is a heartfelt polemic, told from the side of the public good. It is a remarkable trajectory for one scientist, from tracing divisions of worm neuroblasts to managing hundreds of scientists and tens of millions of dollars to finally waging and winning a high-profile battle to keep the results in the public domain.

The founder of the modern *C. elegans* field, the iconoclastic, unconventional, and ever-quotable Sydney Brenner, is best encountered in his own words, in a series of conversations with Lewis Wolpert collected as ***My Life in Science*** (2001, eds. Errol Friedberg and Eleanor Laurence). Brenner, like Crick (and despite the office sign), is a voracious reader. Two of his favorites are the autobiographies of the geneticist and nematode neuroanatomist Richard Goldschmidt and the physicist Max Born (Delbrück's thesis advisor). *My Life in Science* is now out of print but is partly subsumed in Friedberg's recent ***Sydney Brenner: A Biography*** (2010). It is clearly a challenge to encompass Brenner's myriad activities, opinions, and schemes in a single volume, and if Friedberg's account is a little low on analysis or penetrating psychological insights, the chronicle itself is fascinating.

I end these recommendations with a brief look at another important strand in modern genetics, chromosome structure, and dynamics. One of the towering figures here is Barbara McClintock, and I recommend the excellent biography ***The Tangled Field: Barbara McClintock's Search for the Patterns of Genetic Control*** (Nathaniel Comfort, 2003), preferably to be read in tandem with Evelyn Fox Keller's ***A Feeling for the Organism*** (Jane Gitschier's recommendation). Comfort's analysis pushes back at the myth of the marginalized “intuitive” female scientist (partly a creation of Fox Keller and perhaps partly of McClintock herself). In place of the myth, McClintock seems all the more awe-inspiring: a brilliant, highly analytical mind following a profoundly unconventional path.

McClintock was one of the first to recognize the special properties of chromosome ends, or telomeres. It was not until decades later that Elizabeth Blackburn and others, working in *Tetrahymena*, made the critical breakthroughs in defining telomere structure and synthesis. Catherine Brady's biography ***Elizabeth Blackburn and the Story of Telomeres*** (2007) gives a careful and accurate account of the key experiments, showing how Blackburn alternated between bold imaginative leaps and rigorous tests of these predictions. Despite her love of life in the lab, Blackburn was willing to take on significant public service; her bruising experience on the highly politicized President's Council for Bioethics is an intriguing cautionary tale. This biography appeared before Blackburn (with Carol Greider and Jack Szostak) was awarded the Nobel Prize and avoids some of the hagiographical tendencies of other laureate biographies.

Anyone reading the above in search of guidance on ”how to win the Nobel Prize” (to quote the title of an excellent memoir by J. Michael Bishop) may be disappointed. Each protagonist took their own uniquely tortuous path to discovery and fame. Some were academic prodigies (Brenner), and others were late bloomers (Crick) or unfocused (Monod). Some were politically committed (Brenner, Luria, and Monod), while others stayed aloof in the ivory tower. They were all ambitious, but for many the existential crisis of war may have given them a sense of the commitment needed to take on the big problems. Chance and circumstance were important, yet luck had little do with it: rather, as Pasteur said, chance favored the prepared mind—prepared, that is, to make connections and take imaginative leaps by having maximized the “inductive space” of observations, discussions, and reading.

